# Hepatitis C Virus NS3 Protein Can Activate the Notch-Signaling Pathway through Binding to a Transcription Factor, SRCAP

**DOI:** 10.1371/journal.pone.0020718

**Published:** 2011-06-06

**Authors:** Atsushi Iwai, Tsutomu Takegami, Takuya Shiozaki, Tadaaki Miyazaki

**Affiliations:** 1 Department of Bioresources, Hokkaido University Research Center for Zoonosis Control, Sapporo, Hokkaido, Japan; 2 Medical Research Institute, Kanazawa Medical University, Uchinada, Ishikawa, Japan; Saint Louis University, United States of America

## Abstract

Persistent infections of hepatitis C virus (HCV) are known to be a major risk factor for causing hepatocellular carcinomas. Nonstructural protein 3 (NS3) of HCV has serine protease and RNA helicase domains, and is essential for the viral replication. Further, NS3 is also considered to be involved in the development of HCV-induced hepatocellular carcinomas. In this report, we focus on the function of NS3 protein, and propose a novel possible molecular mechanism which is thought to be related to the tumorigenesis caused by the persistent infection of HCV. We identified SRCAP (Snf2-related CBP activator protein) as a NS3 binding protein using yeast two-hybrid screening, and a co-immunoprecipitation assay demonstrated that NS3 can bind to SRCAP in mammalian cells. The results of a reporter gene assay using *Hes-1* promoter which is known to be a target gene activated by Notch, indicate that NS3 and SRCAP cooperatively activate the *Hes-1* promoter in Hep3B cells. In addition, we show in this report that also p400, which is known as a protein closely resembling SRCAP, would be targeted by NS3. NS3 exhibited binding activity also to the 1449–1808 region of p400 by a co-immunoprecipitation assay, and further the activation of the Notch-mediated transcription of *Hes-1* promoter by NS3 decreased significantly by the combined silencing of *SRCAP* and *p400 *mRNA using short hairpin RNA. These results suggest that the HCV NS3 protein is involved in the activation of the Notch-signaling pathway through the targeting to both SRCAP and p400.

## Introduction

The hepatitis C virus (HCV), a member of the Flaviviridae family, is known as a major risk factor for hepatocellular carcinomas. Infection with HCV frequently becomes a persistent infection and causes chronic hepatitis. During the course of long term HCV infections, chronic hepatitis frequently develops hepatic cancers through hepatic cirrhosis. The HCV has a single positive-stranded RNA as the genome, and initially the viral proteins are synthesized as a single polypeptide, and then the polypeptide is cleaved by the viral and host cellular protease into the mature components of the virus [1]. It has been reported that the viral components, Core [2], [3], NS3 (nonstructural protein 3) [4], NS4B [5], and NS5A [6] independently indicate cell transforming activity, and these viral proteins are considered to be involved in the tumorigenesis caused by HCV infection. One of these proteins, NS3, has two enzymatic functions, serine protease [7]–[9] and RNA helicase [10]. Like other viral proteins, NS3 is known as a multifunctional protein which targets a variety of host factors and modulates its function. For instance, it has been reported that the HCV NS3 protein inhibits the protein kinase A (PKA) [11] and PKC [12] functions, and like other HCV proteins, the Core [13] and NS5A [14], p53 is also targeted by NS3 [4], [15], [16].

The Notch signaling pathway is evolutionarily conserved in many species, and is responsible for cell differentiation and proliferation [17], [18]. Four Notch family genes have been identified in humans, and these genes encode transmembrane receptors which recognize the membrane-associated ligands known as Delta-1 like 1, 3, 4 and Jagged-1, 2. After the ligand binding to the receptor form of Notch, the intracellular domain is released by proteolytic cleavage, and the active form of Notch is translocated into the nucleus where it activates target genes. Aberrant activation of the Notch-signaling pathway was observed in many tumor cell lines, and is also found in cells derived from hepatocellular carcinomas [19]–[22]. However it is known that the Notch-signaling pathway indicates both a transform activity and an anti-tumor activity depending on the status of the cell, in general, it is considered that the Notch-signaling pathway represses cell differentiation and promotes cell mitosis of stem cells [23], [24]. Therefore the Notch-signaling pathway is important for the maintainenance of homeostasis through the normal development of cells, and dysfunction of the molecular mechanism to control the Notch-signaling pathway is closely related to tumorigenesis.

Some reports have suggested that abnormality of the Notch-signaling pathway contributes to tumorigenesis of hepatocellular carcinomas [20]–[22], however, the effects of HCV viral infection on the Notch-signaling pathway have not been clarified. In this report, we focus on a NS3-binding molecule, and propose a novel possible mechanism for tumorigenesis caused by the persistent infection of HCV. We demonstrate that the HCV NS3 protein binds to SRCAP (Snf2-related CBP activator protein) and is involved in the activation of the Notch-signaling pathway. The SRCAP was found as a candidate molecule for an NS3 binding protein by yeast two-hybrid screening which we have previously performed using NS3 as a bait [25]. Originally, SRCAP was identified as a binding partner of CBP (CREB binding protein) belonging to the SNF2/SWI2 protein family, and acting as a transcriptional activator [26]. Previously a report has indicated that SRCAP is a mammalian homologue of the *domino* gene of *Drosophila melanogaster* and is involved in the activation of Notch-mediated transcription [27]. The amino acid sequence of SRCAP has a significant similarity with p400 which is known to be targeted by an adenovirus oncoprotein, E1A [28]. Also, p400 is known to be a mammalian homologue of the *domino* gene, and is involved in the tumorigenesis caused by adenovirus infection. From this, we examined the p400 function in the Notch-signaling pathway, and showed that also p400 is targeted by NS3 and involved in the activation of the Notch-signaling pathway.

## Results

### HCV NS3 protein bind to SRCAP, the transcriptional co-activator

**Figure 1:**
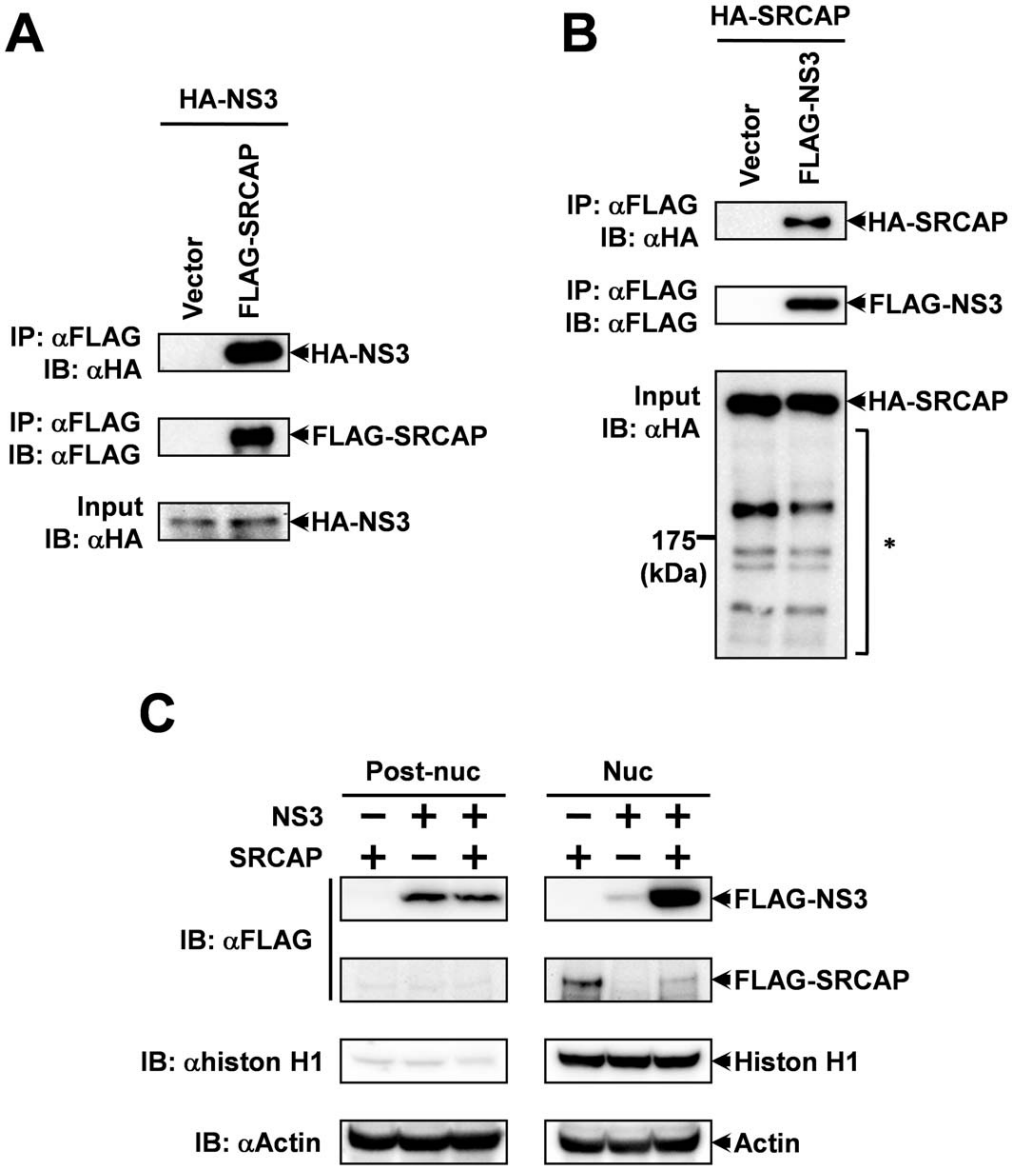
Identification of SRCAP as a host factor which is targeted by HCV NS3. (A, B) HEK293 cells were transfected with the expression vectors for HA-tagged NS3 and FLAG-tagged SRCAP (A), or the expression vectors for HA-tagged SRCAP and FLAG-tagged NS3 (B). After 48 hrs, the cells were harvested and the whole cell lysates were subjected to the immunoprecipitation assay using anti-FLAG M2 agarose (Sigma) as described in the Materials and Methods. The asterisk (*) indicates the bands which are considered to be degradation products of exogenously expressed SRCAP. (C) HEK293 cells were transfected with expression vectors for FLAG-tagged NS3 and SRCAP proteins. After 24 hrs, the cells were harvested, and were fractionated into nuclear and post-nuclear fractions. Anti-histon H1 and anti-actin antibodies were used as a nuclear control and a loading control, respectively.

To understand the molecular function of the HCV NS3 protein in the tumorigenesis caused by HCV infection, we have previously performed yeast two-hybrid screening against the HeLa cDNA library to find NS3 binding proteins [25]. The screening yielded several positive clones considered to be encoding NS3 binding proteins, and one of the positive clones was identified as carrying the SRCAP (Snf2-related CBP activator protein) mRNA. We cloned SRCAP cDNA, and constructed mammalian expression vectors for FLAG and HA-tagged SRCAP, and then the binding activity of NS3 to the SRCAP in mammalian cells was investigated. The FLAG-tagged SRCAP and HA-tagged NS3 were expressed in HEK293 cells and the whole cell extracts of the cells were subjected to a co-immunoprecipitation assay using anti-FLAG M2 agarose. The results indicated that HA-tagged NS3 were co-immunoprecipitated with FLAG-tagged SRCAP (Fig. 1A), whereas no NS3 protein was detected in the immunoprecipitated fraction of the control vector transfected cells. In addition, HA-tagged SRCAP was co-immunoprecipitated with FLAG-tagged NS3 when both proteins were expressed in HEK293 cells (Fig. 1B). These data indicate that the NS3 protein exhibits binding activity to SRCAP in mammalian cells. In addition, some low molecular weight bands, considered to be degradation products of SRCAP, were detected in the lanes of the whole extracts from SRCAP transfected cells, however the patterns of these bands were not significantly changed by expression of NS3. It has been reported that NS4A is needed as a cofactor to fully activate NS3 protease function. However, it is also known that NS3 expressed alone indicates weak protease activity [29]–[31]. Therefore, the data suggest that SRCAP is not a substrate of the proteolysis by NS3.

SRCAP is known to be a transcriptional factor, and is considered to be localized in nucleus [26]. On the other hand, previous reports showed that subcellular localization of NS3 is mainly observed in cytoplasm, and is especially accumulated to the endoplasmic reticulum (ER) [32. 33]. From these findings, we thought the possibility that subcellular localization of NS3 is affected by the expression of SRCAP. To examine this possibility, we separated the HEK293 cells which were transfected with the expression vectors for FLAG-tagged NS3 and SRCAP proteins into nuclear and post-nuclear fractions, and analyzed these fractions by Western blotting. As shown in Fig. 1C, the results show that nuclear-localized NS3 protein was significantly increased by the co-expression of SRCAP. These observations suggested the possibility that a function of SRCAP is modulated by the NS3 in nucleus.

### HCV NS3 protein enhances both Notch1- and Notch3-induced *Hes-1* promoter activation cooperatively with SRCAP

The SRCAP protein has been found to be a transcription factor belonging to the SNF/SWI family [26]. It has been reported that SACAP is a mammalian homologue of the *domino* gene product of *Drosophila melanogaster*, and that it is responsible for activating the Notch-signaling pathway [27]. From these findings, we thought that NS3 might affect the Notch-signaling pathway through binding to SRCAP. To investigate the effects of the NS3 protein against SRCAP mediated Notch-signaling activation, we performed a reporter gene assay.

**Figure 2:**
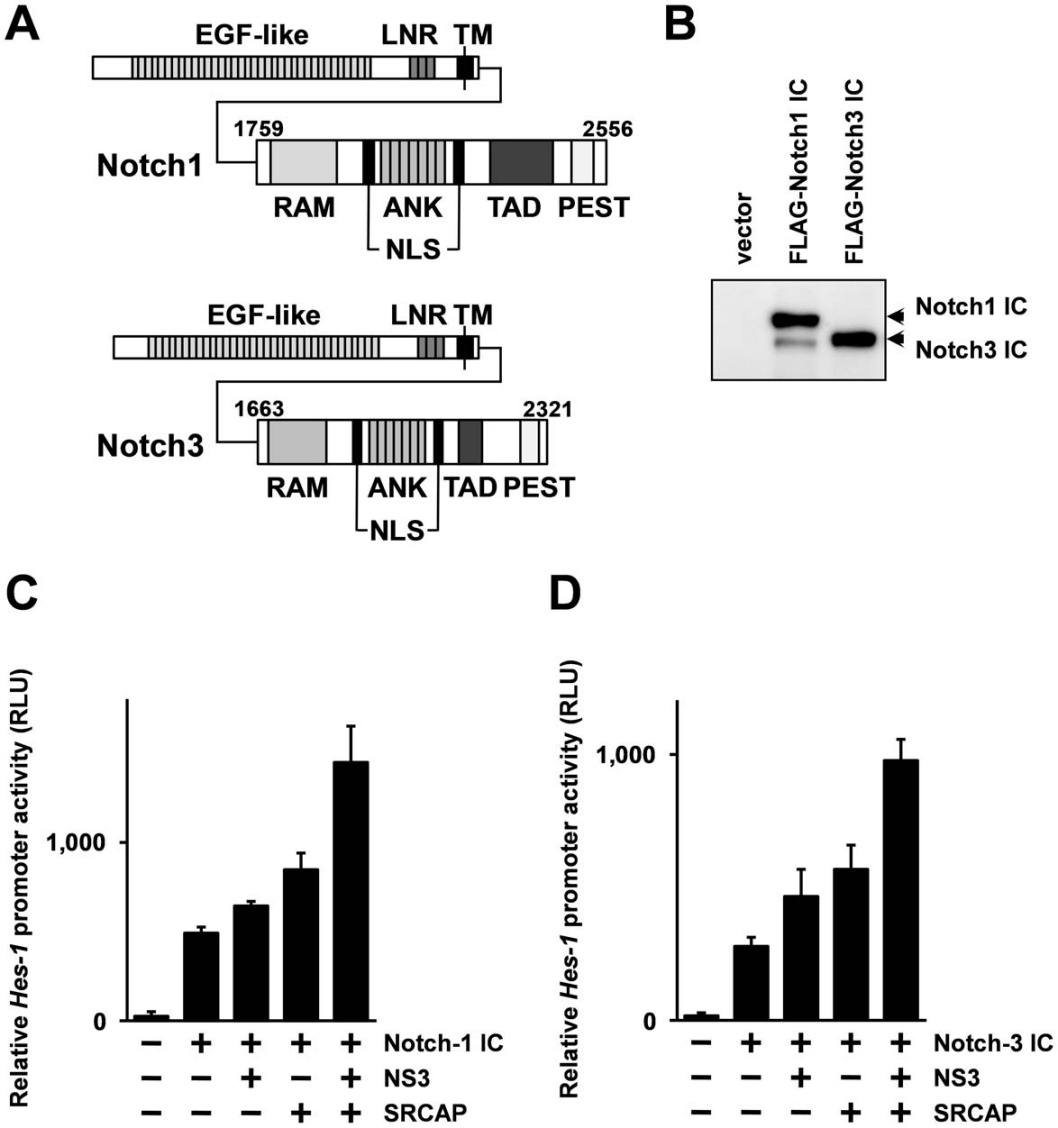
NS3 and SRCAP cooperatively activate Notch-mediated transcription of *Hes-1* promoter. (A) Schematic illustration of domain structure of human Notch1 and Notch3. The amino acid positions of the intracellular domains of Nocth1 and Notch3 are indicated in the figure. EGF-like, EGF-like domain; LNR, LIN12/Notch repeats; TM, transmembrane region; RAM, RAM (RBP-jk-associated molecule) domain; NLS, nuclear localization signal; ANK, ankyrin repeat; TAD, transactivation domain; PEST, PEST (proline (P)-, glutamic acid (E)-, serine (S)- and threonine (T)-rich) sequence. (B) Protein expression analysis of FLAG-tagged intracellular (IC) domain of Notch1 and Notch3. The expression vector for FLAG-tagged Notch1 IC or Notch3 IC was transfected into HEK293 cells. After 24 hrs, the whole cell extracts were subjected to the immuno-blotting analysis using anti-FLAG antibody. (C, D) The expression vectors for FLAG-tagged Notch1 IC (C), or Notch3 IC (D) and the expression plasmids for FLAG-tagged NS3 and SRCAP were transfected into Hep3B cells together with reporter constructs, pGL4.20 *Hes-1* pro and pcDNA3.1 (+) *Rluc*. After 48 hr, the luciferase activities were quantified by luminometer. The relative Hes-1 promoter activities are expressed in relative luminescence units (RLU) normalized by *Renilla* luciferase activities. Error bars indicating the standard deviations were calculated from at least three independent experiments.

It has been reported that aberrant expression of Notch3 is found in many hepatocellular carcinoma cells, as frequently as that of Notch1 [19]–[22]. Thus, we initially constructed expression vectors for the intracellular region of human Notch1 (Notch1 IC) and Notch3 (Notch3 IC). Although the functional differences between Notch1 and Notch3 were not established, as shown in Fig. 2A, in the structural features, Notch3 has a shorter extracellular EGF-like domain and transactivation domain than Notch1 [17], [18]. Both Notch1 and Notch3 have an extracellular domain for recognition of the stimulation by the ligands, and the intracellular region contains nuclear localization signals and a transactivation domain. By stimulation of the ligand, the intracellular region is cleaved and translocated to the nucleus, and then activates the target genes. Therefore, the intracellular region of Notch is known to act as the active form of Notch. Western blotting analysis of the expression of Notch1 IC and Notch3 IC in HEK293 cells indicated that both of these proteins were well expressed and exhibited similar expression efficiencies (Fig. 2B).

Next, we investigated the effects of the NS3 expression on the Notch-signaling pathway using reporter gene assay using a reporter plasmid containing the firefly luciferase gene under the control of *Hes-1* (hairy and enhancer of split 1) promoter; *Hes-1* is known as a target gene activated by Notch, and a reporter construct using *Hes-1* gene promoter is widely used for monitoring activation of the Notch-signaling pathway [34]. The reporter plasmids, NS3 and SRCAP expression vectors were transfected into Hep3B cells together with the plasmid expressing Notch1 IC or Notch3 IC. After 48 hrs, the activation of the *Hes-1* promoter was monitored by the luciferase activity using a luminometer. The results show that activation of *Hes-1* promoter by Notch1 IC was significantly enhanced by overexpression of SRCAP and NS3 (Fig. 2C). Comparable results were obtained by the Notch3 IC mediated activation of *Hes-1* promoter. As shown in Fig. 2D, activation of *Hes-1* promoter mediated by the expression of Notch3 IC was significantly increased by the combined expression of NS3 and SRCAP. These results indicate that NS3 and SRCAP proteins cooperatively activate Notch-signaling pathway in a transcription level.

### Protease activity of NS3 is not required for the activation of Notch signaling pathway by NS3

**Figure 3:**
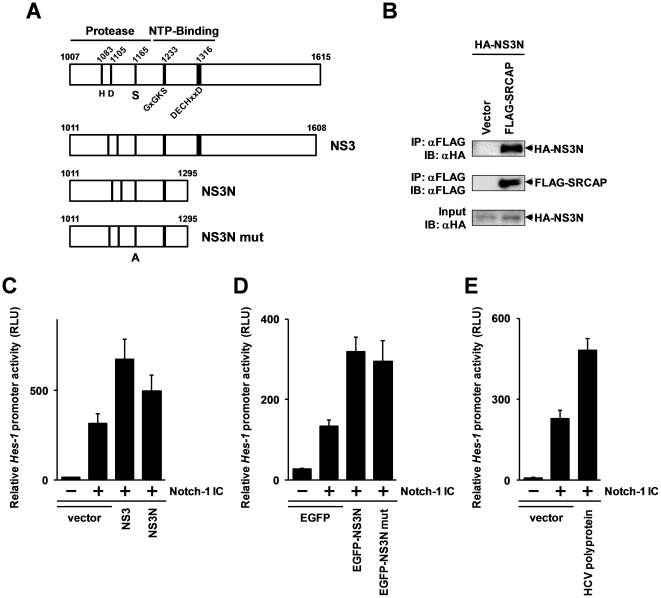
Protease activity of NS3 is not required for activation of Notch-signaling pathway. (A) Schematic diagram of the NS3 deletion mutant and its protease deficient mutant used in this study. The amino acid residues of the serine protease catalytic triad and the conserved motifs of helicases are indicated in the figure. (B) The expression vector for the HA-tagged N-terminal protease region of NS3 and FLAG-tagged SRCAP were transfected into HEK293 cells. After 48 hrs, the whole extracts from the cells were immunoprecipitated with anti-FLAG agarose (Sigma), and the immunoprecipitated fractions were analyzed by immunoblotting. (C) The expression vector for FLAG-tagged NS3 or the N-terminal portion of NS3 and the expression plasmid for Notch1 IC were transfected into Hep3B cells together with pGL4.20 Hes-1 pro and pcDNA3.1 (+) Rluc. After 48 hrs, the luciferase activities were quantified by luminometer. (D) The EGFP-fused N-terminal protease region of NS3 or its protease deficient mutant was transfected into Hep3B cells together with the Notch1 IC expression vector, pGL4.20 Hes-1 pro and pcDNA3.1 (+) Rluc. After 48 hrs, luciferase activities were measured by luminometer. (E) The expression vectors for full-length HCV polyprotein and for Notch1 IC were transfected into Hep3B cells together with the reporter gene constructs. After 48 hrs, luciferase activities were quantified. The relative *Hes-1* promoter activities are expressed in RLU normalized by *Renilla* luciferase activities. Error bars indicating the standard deviations were calculated from at least three independent experiments.

As shown in Fig. 3A, NS3 has two catalytic domains, one is a serine protease domain which is located at the N-terminus one third of NS3, and other is an RNA helicase domain at the C-terminus. A previous report indicates that the transforming activity of NS3 can be assigned to the N-terminus protease region [4], and this region was also used as the bait for our previously performed yeast two-hybrid screening [25]. To verify this, we investigated whether the N-terminal portion of NS3 (the 1011 to 1295 amino acid region of the HCV type 1 b polyprotein) binds to SRCAP by a co-immunoprecipitation assay. The results showed that the N-terminal portion of the NS3 protein also exhibits binding activity to SRCAP (Fig. 3B), in agreement with the result of the yeast two-hybrid screening.

Next, we investigated function of the N-terminal portion of NS3 for the Notch-mediated transcription of the *Hes*-1 promoter. The results show that although the activity of the N-terminal portion of NS3 was weaker than the full-length NS3, the N-terminal portion of NS3 also activated the Notch1 IC-mediated transcription of *Hes*-1 promoter (Fig. 3C). The result suggests that function of NS3 for activation of Notch-signaling is assigned in N-terminal protease region of NS3. Thus, we investigated whether the protease activity of NS3 is involved in the activation of the Notch-mediated transcription. The N-terminal portion of NS3 or of the protease-dead mutant NS3 (Fig. 3A) [7] fused with EGFP (enhanced green fluorescent protein) was transfected into Hep3B cells, and the reporter gene activities under the control of *Hes*-1 promoter were monitored. The results show that both the N-terminal portion of NS3 and its protease-dead mutant activated the Notch1 IC induced transcription of the *Hes*-1 promoter (Fig. 3D). These data suggest that the protease activity of NS3 is not required for the activation of Notch-mediated transcription.

Finally, to investigate whether the NS3 function for activation of Notch-signaling pathway is also functional under the condition of which the other HCV proteins exist, we performed the reporter gene assay using expression vector for full-length HCV polyprotein. The results show that Notch1 IC-mediated activation of *Hes-1* promoter was enhanced by the expression of the full-length HCV polyprotein (Fig 3E).

### NS3 also binds to the fragment of p400 which is homologous to the NS3-binding region of SRCAP

**Figure 4:**
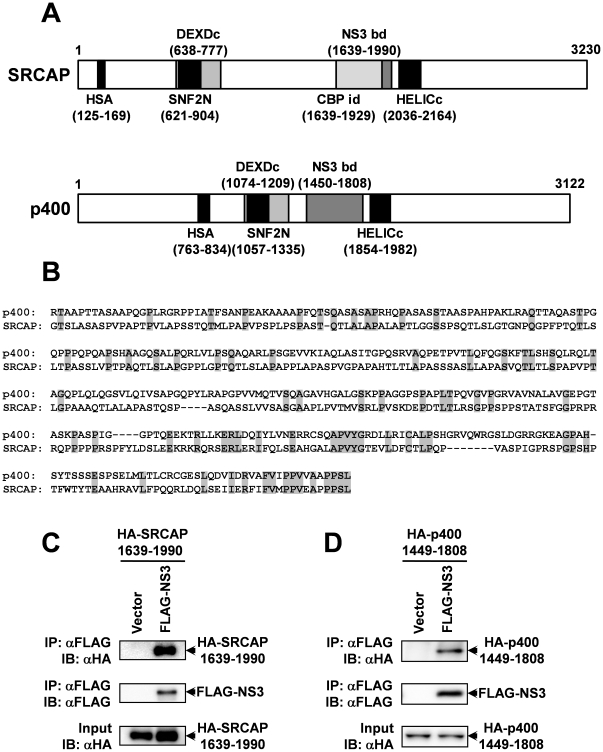
NS3 binds to the amino acid region 1449–1808 of the p400 protein. (A) Schematic illustration of domain structure of human SRCAP and p400. The domain structures predicted by using conserved domain database (CDD) are indicated in the figure. HAS, helicase-SANT-associated (HSA) domain; SNF2N, SNF2 family N-terminal domain; DEXDc, DEAD-like helicases superfamily domain; CBPid, CBP interaction domain; NS3 bd, HCV NS3 protein binding domain; HELICc, Helicase superfamily c-terminal domain. (B) Amino acid sequence alignment of the NS3-binding region of SRCAP and its equivalent region in p400. The NS3 binding region of SRCAP (amino acid position: 1639–1990) was aligned with the p400 sequence using CLUSTAL W [62]. Identical amino acids are shown in gray. (C, D) The expression vector for HA-tagged SRCAP (1639–1990) (C) or p400 (1449–1808) (D) was transfected into HEK293 cells together with the expression plasmid for FLAG-tagged NS3. After 24 hrs, the cells were harvested, lysed, and then subjected to the co-immunoprecipitation analysis using anti-FLAG M2 agarose (Sigma).

Sequence analysis showed that the positive clone obtained by the yeast two-hybrid screening encodes the 1639–1990 amino-acid region of SRCAP (Fig. 4A). To investigate whether this region of SRCAP is responsible for the binding to NS3, we constructed a plasmid to express this region of SRCAP and performed a co-immunoprecipitation assay. The results show that HA-tagged SRCAP 1639–1990 was co-immunoprecipitated with FLAG-tagged-NS3 (Fig. 4C), suggesting that this region which almost fully overlaps the previously reported CBP binding site of SRCAP (amino acid position 1649–1988; note that the position of the amino acid has been changed from the previously reported numbers, because the sequence of SRCAP in the database has been updated.) [26] is responsible for binding to NS3.

There is a significant sequence similarity of SRCAP with p400, which is known as a transcriptional factor binding to the adenovirus E1A oncoprotein [27], [28], and sequence analysis showed that the region homologous to the p400 for the NS3 binding region of SRCAP can be assigned to amino acid position 1450–1808 (Fig. 4A and 4B). We constructed a HA-tagged expression vector for this region by cloning, and performed a co-immunoprecipitation assay to investigate the binding activity of NS3 to this region. The results showed that NS3 is also able to bind to this region of p400 (Fig. 4D). The data suggest that p400 must be considered a candidate for host-cellular molecules targeted by NS3 as well as by SRCAP.

### Gene specific silencing of SRCAP and p400 mRNAs by short hairpin RNAs inhibits the NS3-mediated activation of *Hes-1* promoter

**Figure 5:**
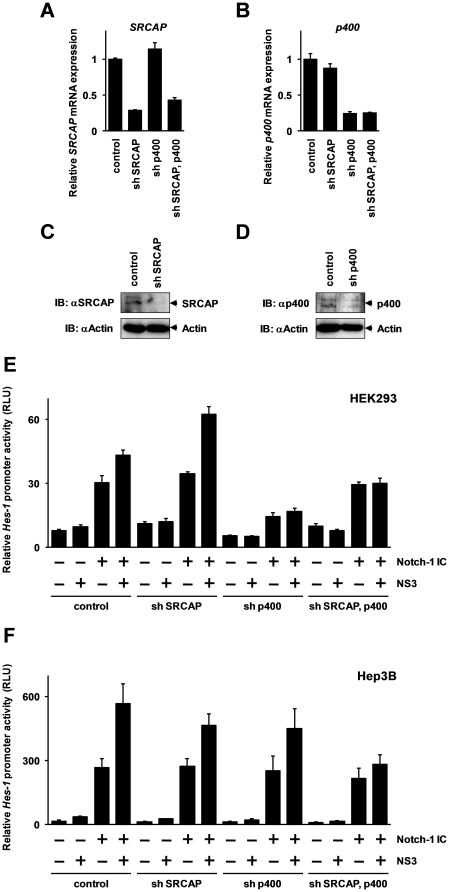
Combinational knockdown of *SRCAP* and *p400* inhibits the NS3 function for the Notch-signaling pathway. (A, B) Analysis of the gene silencing efficiency of the shRNA expression vectors used in this study. The *SRCAP* or *p400* targeted shRNA expression vector was transfected into HEK293 cells. The total amount of the expression vectors for shRNA in each transfections were equalized using the control shRNA expression vector. After 48 hrs, the cells were harvested, and the expression levels of *SRCAP* (A) and *p400* (B) mRNAs were quantified by real-time RT-PCR. Data represent relative expression amount compared with that of non-specific shRNA-transfected cells. Error bars indicate standard deviations calculated by at least three independent experiments. (C, D) The protein expression levels of SRCAP and p400 in shRNA-transfected HEK293 cells were analyzed by Western blotting using specific antibodies against SRCAP and p400. Actin was used for loading control. (E, F) The HEK293 (E) or the Hep3B (F) cells were transfected with expression vectors for *SRCAP* and *p400* specific shRNAs. After 48 hrs, the cells were transfected with reporter gene constructs together with FLAG-tagged Notch1 IC and NS3 expression vectors. After an additional 48 hr incubation period of post-transfection, the luciferase activities were measured using a Dual-Glo luciferase assay kit by luminometer. The relative *Hes-1* promoter activities are expressed in RLU normalized by *Renilla* luciferase activities. Error bars indicate standard deviations calculated by at least three independent experiments.

To investigate the effect of *SRCAP* knockdown in relation to the enhancement of Notch-mediated activation of *Hes-1* promoter by NS3, we initially performed the reporter gene assay using short hairpin RNA (shRNA) to specifically silence the SRCAP mRNA expression via RNA interference. However, the knockdown of *SRCAP* mRNA did not significantly affect the activation of the *Hes-1* promoter (data not shown). As shown in Fig. 4D, the results of the co-immunoprecipitation assay showed that NS3 also exhibits binding activity to a partial coding region of p400 which is a protein closely related to SRCAP. This finding suggested the possibility that both SRCAP and p400 are involved in the enhancement of Notch-mediated *Hes-1* promoter activation by NS3. To evaluate this, we constructed a plasmid vector to express shRNA which specifically silences the gene expression of *p400* in addition to the shRNA expression vector for *SRCAP*. These shRNA expression vectors were transfected into HEK293 cells and the expression levels of *SRCAP* and *p400* mRNAs were quantified by real-time RT-PCR to confirm the gene silencing efficiency of these shRNAs expression vectors against endogenously expressed *SRCAP* and *p400* mRNA. The results show that endogenously expressed *SRCAP* and *p400* mRNAs were efficiently repressed by the transfection of the expression vector for *SRCAP* and *p400* specific shRNA (Fig. 5A and 5B). Comparable results were obtained by Western blotting analysis. As shown in Fig. 5C and 5D, the expression of SRCAP and p400 proteins was significantly repressed by the expression of these gene specific shRNAs.

Next, to investigate the effects of gene silencing of *SRCAP* and *p400* on the Notch-signaling pathway, we performed a reporter gene assay. The HEK293 cells were transfected with the shRNA expression vectors. After 48 hrs, the cells were transfected with the reporter gene construct together with Notch1 IC and NS3 expression vectors. After an additional 48 hr of incubation of the post-transfection, the luciferase activities were measured by a luminometer. Unexpectedly, the results show that NS3-mediated activation of *Hes-1* promoter induced by Notch1 IC is enhanced by the knockdown of *SRCAP* mRNA, suggesting that SRCAP negatively regulates Notch1 IC-mediated *Hes-1* promoter activation in HEK293 cells (Fig. 5E). These results were contradictory to the results of the reporter gene assay indicating the effect of the SRCAP overexpression in Hep3B cells (Fig. 2C). In addition, the Notch1 IC-induced *Hes-1* promoter activations were strongly repressed by the knockdown of *p400* mRNA, suggesting that p400 is predominantly responsible for activation of the Notch-signaling pathway in HEK293 cells. The mammalian cell line, HEK293 has been established from a transformed cell by the integration of adenovirus type 5 DNA into the genome of human embryonic kidney cells [35], and is constitutively expressing adenovirus E1A protein. Since it is known that SRCAP and p400-mediated transcriptions are modulated by E1A [26], [28], it is likely that the adenovirus E1A protein expressed in HEK293 cells would affect the results. Based on this hypothesis, Hep3B cells were used to re-examine the effects of the gene silencing of *SRCAP* and *p400* mRNA on the Notch-signaling pathway. The results indicate a slight (but not statistically significant) inhibition of the NS3 function to the *Hes-1* promoter activation by the independent knockdown of *SRCAP* or *p400* mRNA (Fig. 5F). In addition, the combined knockdown of *SRCAP* and *p400* mRNAs statistically significantly (P<0.01) inhibited the NS3-mediated *Hes-1* promoter activation. These results indicate that both SRCAP and p400 are involved in the NS3-mediated activation of *Hes-1* promoter.

## Discussion

In this report, we demonstrated that HCV NS3 protein can bind to a host-cellular transcriptional factor, SRCAP, and enhance the Notch-mediated transcriptional activation of *Hes*-1 promoter cooperatively with SRCAP by the overexpression. However it is not clarified whether the NS3-mediated activation of Notch-signaling is also functional in HCV-infected cells, and further investigations under more physiological conditions are required to confirm the significance of of our findings on the life cycles of HCV. Nevertheless, as discussed below, at least the Notch-signaling seems to be activated in the HCV patient's liver, and is thought to be involved in the tumorigenesis caused by the persistent infection of HCV.

A previous report has demonstrated that the expressions of Notch1 and its ligand Jagged-1 were increased in hepatocytes after partial hepatectomy [36]. The study here indicates the importance of Notch-signaling in liver regeneration, and suggests the possibility that tissue damage to the liver caused by the infection of HCV activates Notch-signaling. It has been reported that the Notch-signaling pathway exhibits both pro-tumor and anti-tumor activity [23], [24]. Although increments in Notch expression is frequently observed in hepatocellular carcinoma cells [19]–[22], Notch also exhibits inhibitory activity to cell proliferation of hepatocellular carcinomas [37]. In addition, Notch exhibits both pro-apoptotic and anti-apoptotic functions towards hepatocellular carcinoma cells [38], [39]. Generally, the Notch-signaling pathway is responsible for maintaining cells at the proliferative and undifferentiated states. Therefore, whether Notch promotes or suppresses tumorigenesis is thought to be mostly dependent on the status of the cells. Like Notch-signaling indicates invertible functions dependent on the status of the cells, NS3 exhibits not only the function to promote cell proliferation [40] and to inhibit induction of apoptosis [16], but also activity for induction of apoptosis [41]. The NS3 function for activation of the Notch-signaling pathway may provide a part of the reason for these countervailing functions of NS3.

Constitutive activation of Notch-signaling is frequently observed in human hepatocellular carcinoma and is also found in the carcinomas caused by HCV infection [20], [21]. The activation of Notch-signaling induces expression of its membrane-bound ligands known as Delta-1 like 1, 3, 4 and Jagged-1, 2, and then, these expressed ligands stimulate Notch receptor expressed on the surface of other cells by cell-to-cell contact [17], [18].This activation loop of Notch-signaling may have some advantages for the effective replication of HCV through the activation of cell proliferation of HCV-infected cells, and lead to constitutive activation of Notch during the course of tumor development.

The co-immunoprecipitation assay using partial coding regions of SRCAP showed that the binding region of NS3 on SRCAP is assigned to a region almost overlapping with the CBP-binding region of SRCAP (Fig. 4A and 4C). Since interaction with CBP is crucial for the transcriptional activation function of SRCAP [26 42], NS3 may be involved in recruitment of CBP/p300 to SRCAP, or stabilization of CBP/p300 and SRCAP complexes. In addition, the study here demonstrates that NS3 also binds to the partial coding region of p400 which is homologous with the NS3 binding region of SRCAP (Fig. 4D). Although whether NS3 is actually binding to p400 is not clarified, the gene silencing experiment data here using *SRCAP* and *p400* specific shRNAs demonstrates that both SRCAP as well as p400 are involved in NS3-mediated activation of the Notch-signaling pathway (Fig. 5). Both SRCAP and p400 are known to be p300/CBP binding proteins [26], [28] and previous reports have demonstrated that p300/CBP is important for activation of the Notch signaling pathway [43], [44]. Therefore it would be quite possible that both SRCAP and p400 are involved in the activation of the Notch-mediated transcription.

**Figure 6:**
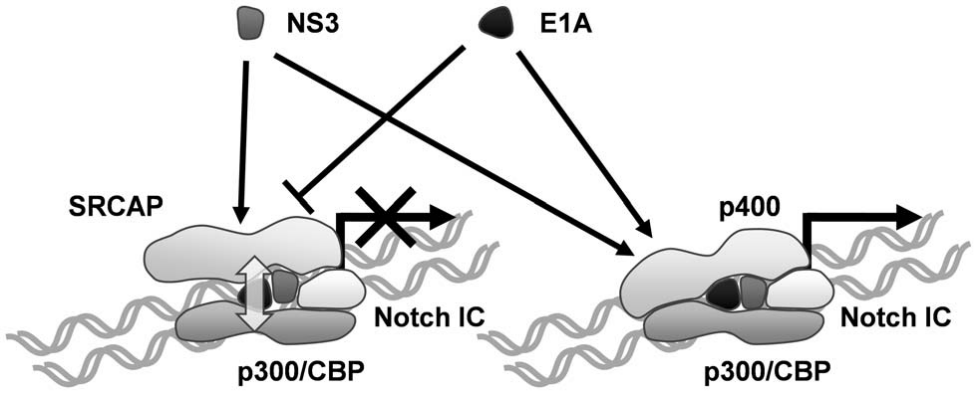
Proposed mechanism of HCV NS3 and adenovirus E1A in the Notch-mediated transcription. The HCV NS3 protein activates both the SRCAP and p400 functions for the Notch-mediated transcription, whereas adenovirus E1A protein activates only the p400 function. Since E1A disrupts the interaction of p300 and p400, enhancement of SRCAP-mediated transcriptional activation of Notch target genes by NS3 is inhibited by the competition with the E1A function. The NS3 function for p400-mediated transcriptional activation is not competitive with the E1A function. Thus an additional expression of NS3 enhances the E1A function for increments in the p400-mediated transcriptional activation.

Originally, p400 has been identified as an adenovirus E1A-binding molecule and is considered to be involved in the tumorigenesis of adenovirus infections [28]. It is known that E1A also binds to p300 and other host-cellular transcriptional factors, and is thought to be involved in transcriptional regulation by formation of the complex with these factors [45], [46]. At the same time, the previous reports have demonstrated that SRCAP-mediated transcriptional activation is inhibited by E1A through the prevention of the interaction of SRCAP and CBP [26], [47]. Taken together, these findings suggest the possibility that E1A plays a role different from NS3 in the activation of Notch-mediated transcription through the modulation of the transcriptional function of SRCAP and p400. The results of the reporter gene assay using HEK293 cells suggest a distinct function of E1A on SRCAP and p400-mediated activation of Notch-signaling (Fig. 5E). The observations indicate that the Notch1 IC-mediated activation of *Hes-1* promoter was strongly suppressed by the knockdown of *p400*, while the knockdown of *SRCAP* resulted in no such effect. Here, the enhancement of activation of *Hes-1* promoter by NS3 is increased by the knockdown of *SRCAP* mRNA. A mechanism to explain these phenomena is indicated in Fig. 6. The HCV NS3 protein activates the transcriptional activator function of both SRCAP and p400, whereas the adenoviral E1A protein activates only the function of p400, but inhibits the SRCAP-mediated transcriptional activation as previously reported [26], [47]. The functions of NS3 and E1A in the modulation of a transcriptional activator function of SRCAP appear to act in a competitive manner, while at the same time NS3 and E1A are thought to be regulating the p400 function in a coordinative manner. Therefore, the SRCAP dependent activation of Notch1 IC-mediated transcription of *Hes-1* promoter is repressed by the competition of the NS3 and E1A functions in the *p400* knockdown HEK293 cells, while a p400 dependent activation of Notch1 IC is coordinately increased by NS3 and E1A proteins in the *SRCAP* knockdown cells (Fig. 5E). In addition, the effects of the knockdown of p400 were partially restored by the additional knockdown of SRCAP mRNA. These results would indicate that the molecular ratio of SRCAP and p400 is important for the activation of Notch-mediated transcription by E1A.

It has been reported that knockdown of SRCAP mRNA represses replication of HCV [48]. This systematic RNAi screening study indicated that about 4.9 fold decrement of the HCV replication and about 1.4 fold decrement of the RNA replication of HCV replicon were observed by the knockdown of SRCAP. Although the dependence on Notch-signaling pathway in this phenomenon is not clarified, the report demonstrated the significance of SRCAP on the effective replication of HCV. On the other hand, a previous report has demonstrated that SRCAP is also targeted by the NS5A protein of HCV [49]. That report indicated that NS5A exhibits binding activity to SRCAP and cooperatively inhibits transcriptional activation of p21 promoter which is a well known target gene regulated by p53. Although the NS5A function for modulating Notch-signaling through the binding to SRCAP has not been clarified, the results of reporter gene assay using expression vector for full length HCV polyprotein indicate that at least NS3-mediated activation of Notch-signaling seems to be not inhibited by the expression of NS5A (Fig, 3E).

Thus far, the focus here has been on the SRCAP function for activation of Notch-mediated transcription, and it has been possible to show the potential of NS3 for the enhancement of SRCAP-mediated activation of the Notch-signaling pathway. However, SRCAP is also known to be responsible for the regulation of other transcriptional factors. In addition to Notch, it may be necessary to analyze the function of SRCAP in the regulation of p53 for a further understanding of the tumorigenesis caused by HCV infections. Transcription of p53 target genes is modulated by SRCAP [50] and p400 [51], and also by p300/CBP which is a binding partner of SRCAP, and p400 is important for regulation of the functions of p53 [52]–[54]. Moreover, p53 is known to be a host-cellular molecule targeted by HCV Core protein [13]. Therefore the function of NS3 in the modulation of SRCAP-mediated transcriptional activation is thought to be affected by the function of Core protein in addition to that of NS5A. Further investigation to analyze the functional relationship of NS3 and the other HCV molecules in the modulation of SRCAP and SRCAP-related molecules will be necessary for a fuller understanding of the detailed molecular mechanism of tumorigenesis caused by HCV infections.

## Materials and Methods

### Cell culture and transfection

The human embryonic kidney cell line HEK293 [35] and the human hepatocellular carcinoma cell line Hep3B [55] were maintained in DMEM (Sigma, St. Louis, MO) and RPMI 1640 (Sigma) respectively. Each medium supplemented with 10% FCS, 100 units/ml penicillin and 100 µg/ml streptomycin, and the cells were grown at 37°C with 5% CO_2_.

Transfections were performed using FuGENE HD (Roche, Mannheim, Germany), X-tremeGENE HP (Roche) or Lipofectamine 2000 (Invitrogen, Carlsbad, CA) according to the manufacturer's protocols.

### Vector Construction

The expression vector for full-length HCV polyprotein, pCAG394, was kindly provided by Dr. Hideaki Aizaki (National Institute of Infectious Diseases, Tokyo, Japan) [56], [57].

To construct epitope tagged SRCAP expression vectors, almost the entire SRCAP cording region (accession number: NM_006662; amino-acid position: 198–3230) was amplified by PCR from the HeLa cDNA library using specific sense primer (5′- GATCGGATCCATGGCCAAGGATGTCAGGCA -3′) and antisense primer (5′- GATCGAATTCTCACGTCTTGGCCTTGCGGC -3′). The amplified cDNA was digested with *BamH*I and *EcoR*I sites integrated in the primers, and inserted into the same site of pcDNA3 FLAG and pcDNA3.1 (+) HA (N). The partial coding region of SRCAP (amino-acid position: 1639–1990) was expressed as a dual epitope-tagged protein carrying the myc and HA tags at the N- and C-terminus, respectively. The 1639–1990 amino-acid region of SRCAP was amplified by PCR using sense primer (5′- CTGGATCCGGAACCTCTTTAGCCTCAGCTT -3′) and antisense primer (5′- CAGAATTCCAGGGAAGGGGGAGGTGCCTCC -3′) from the SRCAP cDNA, and inserted at the *BamH*I-*EcoR*I site of pcDNA3.1 (+) MYC-HA. The part of the human p400 coding sequence which is homologous to the SRCAP NS3 binding region (accession number: NM_015409; amino-acid position: 1449–1808) was amplified by RT-PCR using sense primer (5′- CTGGATCCCCCCGGACGGCAGCCCCCACCA -3′) and antisense primer (5′- GGCTCGAGTAGGGACGGGGGTGCTGCCACC -3′) from HEK293 cells, and inserted into the *BamH*I-*Xho*I site of pcDNA 3.1(+) HA (N).

To construct expression plasmids for FLAG- and HA-tagged NS3, the cDNA fragment which encodes NS3 protein (amino-acid position: 1011–1608) derived from the HCV subtype 1 b was amplified by PCR using sense primmer (5′- CGCCGGATCCCCACTTCTAGGACCGGCCGA -3′) and antisense primer (5′- AGGAATTCCTACCACATTTGATCCCACGAT -3′) from the cloned cDNA [58], and inserted into the *BamH*I-*EcoR*I site of pcDNA3 FLAG and pcDNA3.1 (+) HA (N), respectively. The plasmid expressing FLAG-tagged N-terminal portion of NS3 (amino-acid position: 1011–1295) is described in elsewhere, and the coding region of the N-terminal portion of NS3 was inserted into pcDNA3.1 (+) HA (N) for construction of a HA-tagged plasmid. To construct expression plasmids for an EGFP (enhanced green fluorescent protein) fused N-terminal portion of NS3, the *EcoR*I - *BamH*I fragments carrying the NS3 cording region from pGBT/NS3N or pGBT/NS3N/S1165A [25] were inserted into the same site of pEGFP-c2 (Clontech, Mountain View, CA).

The reporter plasmid carrying the promoter region of the murine *Hes-1* gene was constructed as previously described [34], except pGL4.20 (Promega, San Luis Obispo, CA) was used instead of pGL2 Basic. For construction of the vector expressing the constitutive active form of Notch1 and Notch3, the cDNA encoding intracellular domain of human Notch1 (accession number: NM_017617; amino-acid position: 1759–2556) was amplified by RT-PCR using sense primer (5′- CCGGATCCCGCCGGCGGCAGCATGGCCAGC-3′) and antisense primer (5′- CGCTCGAGTTACTTGAAGGCCTCCGGAATG -3′) from K562 cells [59], and the intracellular domain of human Notch3 (accession number: NM_000435; amino-acid position: 1663–2321) was obtained by RT-PCR using sense primer (5′- TGGGATCCATGGTGGCCCGGCGCAAGCGCGA -3′) and antisense primer (5′- CGCTCGAGTCAGGCCAACACTTGCCTCTTG -3′) from HEK293 cells. Each amplified fragment was inserted into the *BamH*I-*Xho*I site of pcDNA3 FLAG.

To construct shRNA expressing vectors silencing the SRCAP and p400 genes, we used previously reported target sequences which are specifically repressed gene expressions of *SRCAP* [60] and *p400* [61]. Double stranded synthetic oligonucleotide were ligated into pSilencer 5.1-U6 Retro (Applied biosystems, Foster City, CA) according to the manufacturer's protocol, and pSilencer 5.1-U6 Retro Scrambled (Applied biosystems) was used for the control vector which is expressing nonspecific shRNA.

### Immunoprecipitation

Expression vectors for epitope-tagged NS3 (1.0 µg) and SRCAP (3.0 µg) or derivatives of these plasmids were transfected into HEK 293 cells. After 24 hrs, cells were harvested and lysed by RIPA buffer (25 mM Tris-HCl [pH 7.5], 150 mM NaCl, 1% NP-40, 1% sodium deoxycholate, 0.1% SDS) supplemented with a protease inhibitor cocktail (Complete Mini; Roche). After the cell debris was removed by centrifugation, the lysates were subjected to immunoprecipitation using anti-FLAG M2 agarose (Sigma). The resins were washed five times with RIPA buffer and eluted binding proteins by boiling for 5 min with 2 x SDS-PAGE gel-loading buffer (125 mM Tris-HCl [pH 6.8], 5% β-mercaptoethanol, 4% SDS, 20% glycerol, 0.01% Bromophenol blue). Subsequently, the eluted proteins were separated by 5% SDS-PAGE, and analysed by Western blotting using specific antibodies recognizing FLAG (Sigma) and HA (Roche) respectively.

### Luciferase assays

The reporter gene constructs and expression vectors indicated in the figures were transfected into Hep3B cells which were seeded onto a 24-well plate. After 48 hrs, the luciferase activities were quantified with a luminometer (Mithras LB940; Berthold, Bad Wildbad, Germany) using Dual-Glo Luciferase Assay System (Promega) in accordance with the manufacturer's instructions. The data represented relative firefly luciferase activity normalized with the *Renilla* luciferase activity of the pcDNA3.1(+) Rluc.

### Real-time RT-PCR

Total RNA was isolated using Trizol (Invitrogen) and treated with RNase free DNase I (Roche), subsequently the isolated RNA was subjected to a random primed reverse transcriptase reaction using ReverTraAce (Toyobo, Osaka, Japan). The expression levels of *SRCAP* and *p400* mRNAs were quantified using SYBR Premix Ex TaqII (Takara, Otsu, Japan) by the Mx3000P quantitative PCR System (Stratagene, La Jolla, CA). Each procedure was performed according to manufacturer's protocol. The quantification data were normalized with the amount of glyceraldehyde-3-phosphate dehydrogenase (*G3PDH*) mRNA, were expressed in relative expression amount compared with that of non-specific shRNA-transfected cells. The following specific primer sets were used in this study: SRCAP sense primer: 5′- CCCCCTGCTTCTCGCCTGGA -3′; SRCAP antisense primer: 5′- ACGCCTCTGGGCCTCTGCAT -3′); p400 sense primer: 5′- AGGCGACAGGGAGAGTCGCA -3′; p400 antisense primer: 5′- GGATGGCTTCAGCCACCGCA -3′; G3PDH sense primer: 5′- CTACTGGCGCTGCCAAGGC -3′; G3PDH antisense primer: 5′- GTGGGTGTCGCTGTTGAAGTC -3′.

### Nuclear extraction and immunoblotting

Nuclear extraction from HEK293 cells was performed using CelLytic NuCLEAR Extraction Kit (Sigma) according to the manufacturer's protocol. Anti-Histone H1 antibody (AbCam, Cambridge, UK) was used as a control of nuclear fraction, and anti-actin antibody (Becton Dickinson, San Jose, CA) was used as a loading control.

HEK293 cells were transfected with shRNA expression vector which specifically targets to SRCAP or p400 mRNA. After 48 hrs, the cells were harvested, and then lysed by lysis buffer (25 mM Tris-HCl [pH 7.5], 150 mM NaCl, 1% NP-40) supplemented with a protease inhibitor cocktail (Complete Mini; Roche). After the cell debris was removed by centrifugation, and the supernatants were subjected to Western blotting analysis. Specific antibodies against SRCAP (Santa Cruz Biotechnology, Santa Cruz, CA) and p400 (Abnova, Taipei, Taiwan) used for detection of endogenously expressed SRCAP and p400 proteins, respectively.

## References

[1] Shimotohno K, Tanji Y, Hirowatari Y, Komoda Y, Kato N (1995). Processing of the hepatitis C virus precursor protein.. J Hepatol.

[2] Ray RB, Lagging LM, Meyer K, Ray R (1996). Hepatitis C virus core protein cooperates with ras and transforms primary rat embryo fibroblasts to tumorigenic phenotype.. J Virol.

[3] Tsuchihara K, Hijikata M, Fukuda K, Kuroki T, Yamamoto N (1999). Hepatitis C virus core protein regulates cell growth and signal transduction pathway transmitting growth stimuli.. Virology.

[4] Sakamuro D, Furukawa T, Takegami T (1995). Hepatitis C virus nonstructural protein NS3 transforms NIH 3T3 cells.. J Virol.

[5] Park JS, Yang JM, Min MK (2000). Hepatitis C virus nonstructural protein NS4B transforms NIH3T3 cells in cooperation with the Ha-ras oncogene.. Biochem Biophys Res Commun.

[6] Gale M, Kwieciszewski B, Dossett M, Nakao H, Katze MG (1999). Antiapoptotic and oncogenic potentials of hepatitis C virus are linked to interferon resistance by viral repression of the PKR protein kinase.. J Virol.

[7] Grakoui A, McCourt DW, Wychowski C, Feinstone SM, Rice CM (1993). A second hepatitis C virus-encoded proteinase.. Proc Natl Acad Sci U S A.

[8] Grakoui A, McCourt DW, Wychowski C, Feinstone SM, Rice CM (1993). Characterization of the hepatitis C virus-encoded serine proteinase: determination of proteinase-dependent polyprotein cleavage sites.. J Virol.

[9] Tomei L, Failla C, Santolini E, De Francesco R, La Monica N (1993). NS3 is a serine protease required for processing of hepatitis C virus polyprotein.. J Virol.

[10] Suzich JA, Tamura JK, Palmer-Hill F, Warrener P, Grakoui A (1993). Hepatitis C virus NS3 protein polynucleotide-stimulated nucleoside triphosphatase and comparison with the related pestivirus and flavivirus enzymes.. J Virol.

[11] Borowski P, Oehlmann K, Heiland M, Laufs R (1997). Nonstructural protein 3 of hepatitis C virus blocks the distribution of the free catalytic subunit of cyclic AMP-dependent protein kinase.. J Virol.

[12] Borowski P, Schulze zur Wiesch J, Resch K, Feucht H, Laufs R (1999). Protein kinase C recognizes the protein kinase A-binding motif of nonstructural protein 3 of hepatitis C virus.. J Biol Chem.

[13] Ray RB, Steele R, Meyer K, Ray R (1997). Transcriptional repression of p53 promoter by hepatitis C virus core protein.. J Biol Chem.

[14] Majumder M, Ghosh AK, Steele R, Ray R, Ray RB (2001). Hepatitis C virus NS5A physically associates with p53 and regulates p21/waf1 gene expression in a p53-dependent manner.. J Virol.

[15] Ishido S, Hotta H (1998). Complex formation of the nonstructural protein 3 of hepatitis C virus with the p53 tumor suppressor.. FEBS Lett.

[16] Kwun HJ, Jung EY, Ahn JY, Lee MN, Jang KL (2001). p53-dependent transcriptional repression of p21(waf1) by hepatitis C virus NS3.. J Gen Virol.

[17] Kopan R, Ilagan MX (2009). The canonical Notch signaling pathway: unfolding the activation mechanism.. Cell.

[18] Zanotti S, Canalis E (2010). Notch and the skeleton.. Mol Cell Biol.

[19] Giovannini C, Lacchini M, Gramantieri L, Chieco P, Bolondi L (2006). Notch3 intracellular domain accumulates in HepG2 cell line.. Anticancer Res.

[20] Cantarini MC, de la Monte SM, Pang M, Tong M, D'Errico A (2006). Aspartyl-asparagyl beta hydroxylase over-expression in human hepatoma is linked to activation of insulin-like growth factor and notch signaling mechanisms.. Hepatology.

[21] Gramantieri L, Giovannini C, Lanzi A, Chieco P, Ravaioli M (2007). Aberrant Notch3 and Notch4 expression in human hepatocellular carcinoma.. Liver Int.

[22] Gao J, Song Z, Chen Y, Xia L, Wang J (2008). Deregulated expression of Notch receptors in human hepatocellular carcinoma.. Dig Liver Dis.

[23] Radtke F, Raj K (2003). The role of Notch in tumorigenesis: oncogene or tumour suppressor?. Nat Rev Cancer.

[24] Dotto GP (2008). Notch tumor suppressor function.. Oncogene.

[25] Iwai A, Hasumura Y, Nojima T, Takegami T (2003). Hepatitis C virus nonstructural protein NS3 binds to Sm-D1, a small nuclear ribonucleoprotein associated with autoimmune disease.. Microbiol Immunol.

[26] Johnston H, Kneer J, Chackalaparampil I, Yaciuk P, Chrivia J (1999). Identification of a novel SNF2/SWI2 protein family member, SRCAP, which interacts with CREB-binding protein.. J Biol Chem.

[27] Eissenberg JC, Wong M, Chrivia JC (2005). Human SRCAP and Drosophila melanogaster DOM are homologs that function in the notch signaling pathway.. Mol Cell Biol.

[28] Fuchs M, Gerber J, Drapkin R, Sif S, Ikura T (2001). The p400 complex is an essential E1A transformation target.. Cell.

[29] Bartenschlager R, Ahlborn-Laake L, Mous J, Jacobsen H (1994). Kinetic and structural analyses of hepatitis C virus polyprotein processing.. J Virol.

[30] Failla C, Tomei L, De Francesco R (1994). Both NS3 and NS4A are required for proteolytic processing of hepatitis C virus nonstructural proteins.. J Virol.

[31] Lin C, Pragai BM, Grakoui A, Xu J, Rice CM (1994). Hepatitis C virus NS3 serine proteinase: trans-cleavage requirements and processing kinetics.. J Virol.

[32] Selby MJ, Choo QL, Berger K, Kuo G, Glazer E (1993). Expression, identification and subcellular localization of the proteins encoded by the hepatitis C viral genome.. J Gen Virol.

[33] Wolk B, Sansonno D, Krausslich HG, Dammacco F, Rice CM (2000). Subcellular localization, stability, and trans-cleavage competence of the hepatitis C virus NS3-NS4A complex expressed in tetracycline-regulated cell lines.. J Virol.

[34] Jarriault S, Brou C, Logeat F, Schroeter EH, Kopan R (1995). Signalling downstream of activated mammalian Notch.. Nature.

[35] Graham FL, Smiley J, Russell WC, Nairn R (1977). Characteristics of a human cell line transformed by DNA from human adenovirus type 5.. J Gen Virol.

[36] Xu HY, Li BJ, Wang RF, Andersson R (2008). Alterations of Notch/Jagged mRNA and protein expression after partial hepatectomy in rats.. Scand J Gastroenterol.

[37] Qi R, An H, Yu Y, Zhang M, Liu S (2003). Notch1 signaling inhibits growth of human hepatocellular carcinoma through induction of cell cycle arrest and apoptosis.. Cancer Res.

[38] Giovannini C, Gramantieri L, Chieco P, Minguzzi M, Lago F (2009). Selective ablation of Notch3 in HCC enhances doxorubicin's death promoting effect by a p53 dependent mechanism.. J Hepatol.

[39] Wang C, Qi R, Li N, Wang Z, An H (2009). Notch1 signaling sensitizes tumor necrosis factor-related apoptosis-inducing ligand-induced apoptosis in human hepatocellular carcinoma cells by inhibiting Akt/Hdm2-mediated p53 degradation and up-regulating p53-dependent DR5 expression.. J Biol Chem.

[40] Fujita T, Ishido S, Muramatsu S, Itoh M, Hotta H (1996). Suppression of actinomycin D-induced apoptosis by the NS3 protein of hepatitis C virus.. Biochem Biophys Res Commun.

[41] Prikhod'ko EA, Prikhod'ko GG, Siegel RM, Thompson P, Major ME (2004). The NS3 protein of hepatitis C virus induces caspase-8-mediated apoptosis independent of its protease or helicase activities.. Virology.

[42] Monroy MA, Ruhl DD, Xu X, Granner DK, Yaciuk P (2001). Regulation of cAMP-responsive element-binding protein-mediated transcription by the SNF2/SWI-related protein, SRCAP.. J Biol Chem.

[43] Oswald F, Tauber B, Dobner T, Bourteele S, Kostezka U (2001). p300 acts as a transcriptional coactivator for mammalian Notch-1.. Mol Cell Biol.

[44] Chen Y, Shu W, Chen W, Wu Q, Liu H (2007). Curcumin, both histone deacetylase and p300/CBP-specific inhibitor, represses the activity of nuclear factor kappa B and Notch 1 in Raji cells.. Basic Clin Pharmacol Toxicol.

[45] Arany Z, Sellers WR, Livingston DM, Eckner R (1994). E1A-associated p300 and CREB-associated CBP belong to a conserved family of coactivators.. Cell.

[46] Lang SE, Hearing P (2003). The adenovirus E1A oncoprotein recruits the cellular TRRAP/GCN5 histone acetyltransferase complex.. Oncogene.

[47] Xu X, Tarakanova V, Chrivia J, Yaciuk P (2003). Adenovirus DNA binding protein inhibits SrCap-activated CBP and CREB-mediated transcription.. Virology.

[48] Randall G, Panis M, Cooper JD, Tellinghuisen TL, Sukhodolets KE (2007). Cellular cofactors affecting hepatitis C virus infection and replication.. Proc Natl Acad Sci U S A.

[49] Ghosh AK, Majumder M, Steele R, Yaciuk P, Chrivia J (2000). Hepatitis C virus NS5A protein modulates transcription through a novel cellular transcription factor SRCAP.. J Biol Chem.

[50] Park JH, Roeder RG (2006). GAS41 is required for repression of the p53 tumor suppressor pathway during normal cellular proliferation.. Mol Cell Biol.

[51] Chan HM, Narita M, Lowe SW, Livingston DM (2005). The p400 E1A-associated protein is a novel component of the p53 –>p21 senescence pathway.. Genes Dev.

[52] Avantaggiati ML, Ogryzko V, Gardner K, Giordano A, Levine AS (1997). Recruitment of p300/CBP in p53-dependent signal pathways.. Cell.

[53] Gu W, Shi XL, Roeder RG (1997). Synergistic activation of transcription by CBP and p53.. Nature.

[54] Lill NL, Grossman SR, Ginsberg D, DeCaprio J, Livingston DM (1997). Binding and modulation of p53 by p300/CBP coactivators.. Nature.

[55] Aden DP, Fogel A, Plotkin S, Damjanov I, Knowles BB (1979). Controlled synthesis of HBsAg in a differentiated human liver carcinoma-derived cell line.. Nature.

[56] Tsukiyama-Kohara K, Kohara M, Yamaguchi K, Maki N, Toyoshima A (1991). A second group of hepatitis C viruses.. Virus Genes.

[57] Aizaki H, Aoki Y, Harada T, Ishii K, Suzuki T (1998). Full-length complementary DNA of hepatitis C virus genome from an infectious blood sample.. Hepatology.

[58] Aizaki H, Harada T, Otsuka M, Seki N, Matsuda M (2002). Expression profiling of liver cell lines expressing entire or parts of hepatitis C virus open reading frame.. Hepatology.

[59] Lozzio CB, Lozzio BB (1975). Human chronic myelogenous leukemia cell-line with positive Philadelphia chromosome.. Blood.

[60] Jha S, Shibata E, Dutta A (2008). Human Rvb1/Tip49 is required for the histone acetyltransferase activity of Tip60/NuA4 and for the downregulation of phosphorylation on H2AX after DNA damage.. Mol Cell Biol.

[61] Gevry N, Chan HM, Laflamme L, Livingston DM, Gaudreau L (2007). p21 transcription is regulated by differential localization of histone H2A.Z.. Genes Dev.

[62] Thompson JD, Higgins DG, Gibson TJ (1994). CLUSTAL W: improving the sensitivity of progressive multiple sequence alignment through sequence weighting, position-specific gap penalties and weight matrix choice.. Nucleic Acids Res.

